# Stabilization of frontal fibrosing alopecia with topical latanoprost monotherapy

**DOI:** 10.1016/j.jdcr.2024.02.015

**Published:** 2024-03-02

**Authors:** Sarah Alenezi, Mariya I. Miteva

**Affiliations:** Dr. Phillip Frost Department of Dermatology and Cutaneous Surgery, University of Miami Miller School of Medicine, Miami, Florida

**Keywords:** frontal fibrosing alopecia, latanoprost, monotherapy, prostaglandin analog, scarring alopecia

## Introduction

Frontal fibrosing alopecia (FFA) is the most common type of scarring alopecia, characterized by symmetric, progressive recession on the frontotemporal hairline and nonscarring irreversible alopecia on the eyebrows and limbs.^1^ It most commonly affects postmenopausal women. The etiology is unknown, and there is no cure. Management typically involves combination therapy with topical or intralesional corticosteroids and anti-inflammatory agents, such as hydroxychloroquine or calcineurin inhibitors, as well as 5-alpha reductase inhibitors, such as dutasteride and topical/oral minoxidil.^2^

Topical latanoprost solution (0.1%) has shown promising results in nonscarring androgenetic alopecia (AGA).^3^ It has also been reported to help stabilize eyebrow hair loss in FFA,^4^ and in our experience, it is an effective treatment option for this condition (authors’ unpublished data). Here, we report a case study demonstrating the potential efficacy of latanoprost (0.005%) as monotherapy applied twice daily for 12 months in stabilizing hair recessing in a patient with recalcitrant FFA.

## Case presentation

A 68-year-old female patient presented to our clinic with an 8-year history of biopsy-proven, progressing, irregular-type FFA for continued management of her alopecia. The patient had a history of severe drug sensitivities to multiple therapeutics, including oral minoxidil, topical pimecrolimus, and tacrolimus. Patch testing had identified several allergens, and she had been instructed to avoid all allergen-containing products. She complied with all the instructions. Long-term therapy with high-potency topical steroids had failed to halt disease progression, and her first-line family history of breast cancer prevented the use of 5-α-reductase inhibitors, such as finasteride. The patient was severely distressed by the progression of her FFA and reported that it was greatly affecting her quality of life.

**Fig 1:**
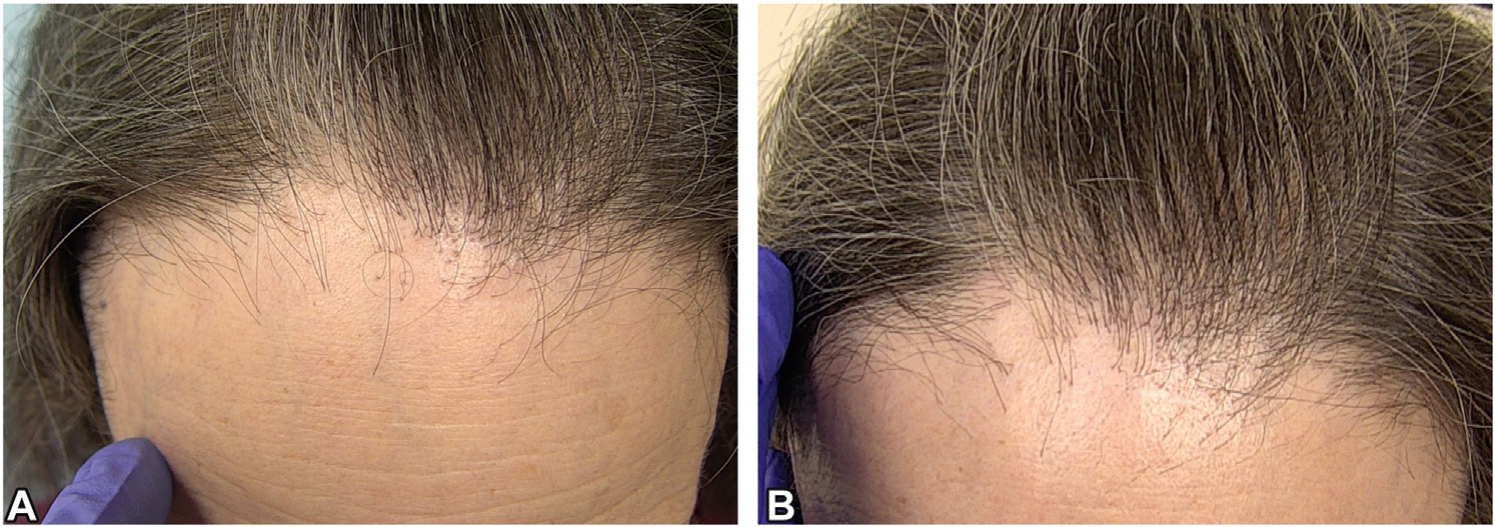
Clinical image of the frontal aspect of the scalp using FotoFinder videodermatoscopy. **A,** Pretreatment: Decreased hair density with hairline reduction. **B,** Posttreatment: Notable absence of progressive hairline reduction or skin thinning, following therapy with topical latanoprost 0.005% solution at the 10-month mark.

**Fig 2:**
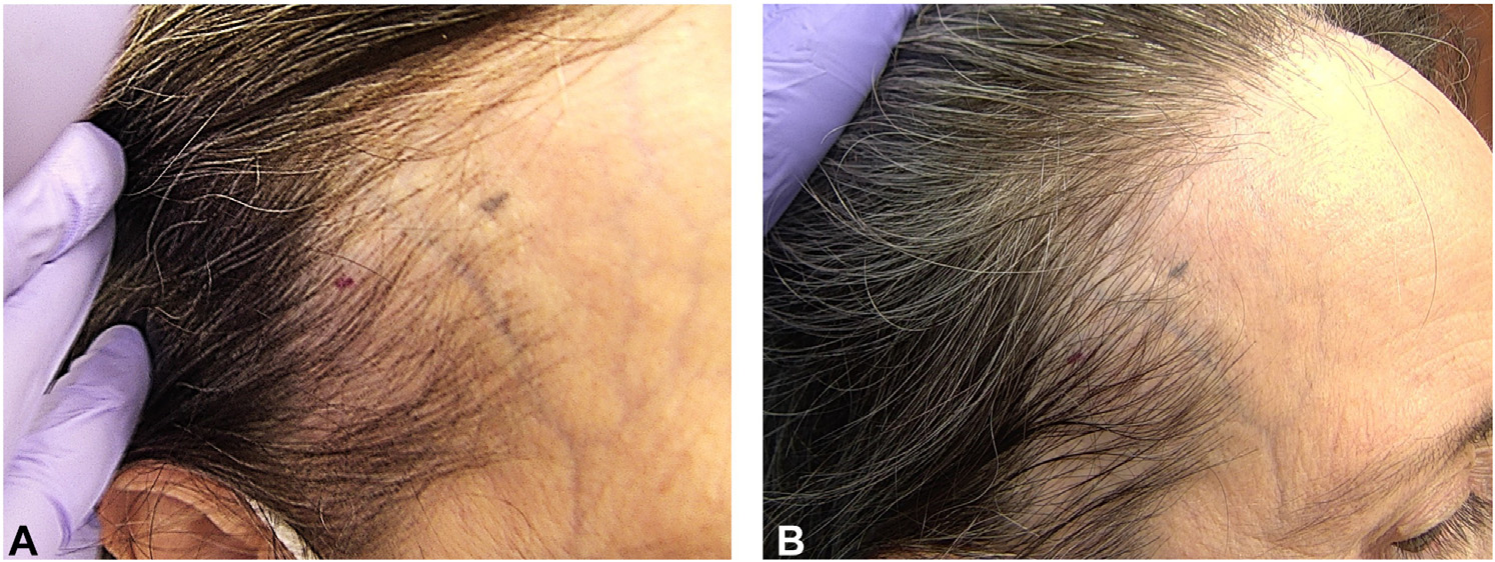
Clinical image of the temporal scalp using FotoFinder videodermatoscopy. **A,** Pretreatment: Decreased hair density with hairline reduction. **B,** Posttreatment: Increase in hair thickness with notable absence of progressive hairline reduction or skin thinning, following therapy with topical latanoprost 0.005% solution at the 10-month mark.

Physical examination revealed a band of decreased hair density on the frontal and temporal aspect of the scalp. The hairline reduction measured 8.0 cm from the glabella and 2.5 cm from lonely hair to the hairline, as compared with 7.0 cm and 2.1 cm, respectively, 4 months ago (Figs 1, *A* and 2, *A*). On FotoFinder videodermoscopy (FotoFinder Systems), a loss of vellus hairs, follicular openings, and peripilar hypopigmentation was observed. Following significant eyebrow hair regrowth with the use of topical bimatoprost (Latisse), the patient was initiated on topical latanoprost 0.005% solution twice daily monotherapy to the affected hairline and 1 inch into the adjacent hair. She reported going through 2.5 mL of latanoprost 0.005% solution every 4 weeks. Ten months later, the patient experienced stabilization of her FFA without any side effects, notably, such as skin atrophy (Figs 1, *B* and 2, *B*). Upon reviewing the trichoscopy images obtained from the same areas of the scalp before and after treatment, we noted that there was no further hairline reduction or increase in the distance from the lonely hair to the hairline. Additionally, on dermatoscopy, we noted an increased thickness in the hair shaft. Therefore, we concluded that the improved hair density is because of increased diameter and pigmentation of the existing hairs and that the patient’s FFA had stabilized.

Ten months later, the patient continues to apply latanoprost 0.005% solution twice daily to her scalp. Her disease remains stable, and she reports a major improvement in her quality of life with halting of the progression of her FFA.

## Discussion

This case highlights the potential of topical latanoprost as monotherapy and an alternative treatment for FFA, offering an alternative and novel option for patients who do not respond to conventional treatments. Our patient was prescribed latanoprost 0.005% applied topically twice daily as an alternative therapy to potentially stabilize hair recession in FFA without any reported side effects. Topical latanoprost could be a valuable alternative for patients who cannot tolerate or use standard treatments for FFA. It is important to consider individual patient factors, such as drug sensitivities and family history, when making treatment decisions for FFA.

Bimatoprost is a Food and Drug Administration-approved prostaglandin-F2α (PGF2α) analog for the treatment of inadequate eyelash growth.^4^ It stimulates melanogenesis, resulting in darker lashes, and increases the size of dermal papilla and hair bulbs, leading to increased lash thickness and fullness.^5^ Prostaglandin analogs, such as latanoprost, are typically used to enhance eyelash growth.^3^^,^^6^ In addition, they have shown promising results in promoting hair regrowth and increasing hair density in unrelated conditions, such as AGA.^7^ In a pilot study, a 24-week therapy with latanoprost 0.1% solution has led to increased hair density and pigmentation compared with placebo in male patients with AGA.^3^

Although prostaglandin analogs are not typically used in the management of FFA, eyebrow improvement with their use has been noticed, as previously mentioned. Topical latanoprost was chosen over bimatoprost because of lower compounding price.

The resultant stabilization of our patient with FFA within 10 months on monotherapy with latanoprost 0.005% solution highlights a potentially new treatment option for this challenging condition. Although further research is needed to explicate the underlying mechanism of the observed clinical response, one can hypothesize that the increase in hair density in unaffected areas can be because of conversion of vellus hair to mature hair, as seen in animal studies.^8^ The PGF2α analogs also harbor anti-inflammatory effects, which could potentially locally halt the inflammation because of FFA.^9^ In addition, considering that PGF2α analogs have been reported to cause periorbital fat atrophy^10^ and that FFA is a condition characterized by inherent epidermal and dermal atrophy, it is imperative to follow patients closely to monitor for signs of atrophy in treated areas. In the case of our patient, no atrophy of treated areas was observed to date, that is, at the 10-month treatment mark. Further research is also needed to understand long-term effects of the use of prostaglandin analogs to treat FFA. Latanoprost and related prostaglandin analogs hold promise as potential treatments for hair loss, it is important to note that the optimal dosage and frequency of treatment with topical latanoprost monotherapy have not been firmly established and require further research.

## Conflicts of interest

None disclosed.
